# A new way of repairing special wounds

**DOI:** 10.1016/j.heliyon.2023.e15755

**Published:** 2023-05-03

**Authors:** Jun Liu

**Affiliations:** Department of Burns, The Gansu Provincial People's Hospital, Lanzhou, China

**Keywords:** Autologous granulation tissue, Autologous thin split-thickness skin, Special wounds

## Abstract

**Purpose:**

Wound repair has always been the basic method of burn surgery, but not all wounds in clinical work can regain both function and appearance. For relatively small wounds with irreversible functional damage accompanied by necrotic bone, joint and tendon exposure, and the wounds in non-functional sites with necrotic bone, tendon exposure and poor surrounding tissue conditions, the value and significance of tissue flap transplantation for wound repair are debatable. This paper discusses a new repair method as a supplementary choice for tissue flap transplantation: autologous granulation tissue and autologous thin split-thickness skin graft, which not only repairs the wound in a simple way but also avoids the cost of tissue flap transplantation.

**Methods:**

A total of 11 patients were collected from June 2019 to July 2022, with a total of 20 exposed wounds of bone, joint and tendon necrosis. During the operation, the necrotic exposed bone tissue and the completely necrotic tendon tissue were removed, and the necrotic soft tissue around the wound was completely excised until the wound appeared bleeding. We cut granulation tissue from other parts of the patient's body with a thickness of about 0.5–0.8 mm, cover the deep wound after thorough debridement with granulation tissue, and transplant autologous thin split-thickness skin to the deep wound covered with granulation tissue. The surgical area was compressed and immobilized.

**Results:**

In 11 patients, 20 wounds were surgically treated, and the wounds healed 15–25 days after the operation, and no bone tissue, joints, or tendons were exposed. No case underwent secondary surgery after surgery. Some wounds were treated with bedside allograft with the consent of the patient due to a small amount of residual granulation wound healing after transplantation.

**Conclusion:**

Using autologous granulation tissue and autologous thin split-thickness skin transplantation to repair some special wounds can not only repair the wounds simply and effectively but also avoid the cost of tissue flap transplantation.

## 1 Introduction

Wound repair has always been a necessary and basic method in the field of surgery, especially in burn and plastic surgery. Autologous skin grafting includes skin graft and cutaneous flap. Skin grafting is suitable for repairing superficial defects in the soft tissue on the body surface. Clinically, cutaneous flap is the best choice for morphological deformities or dysfunctions caused by deep tissue defects such as bone exposure, tendon exposure, and chronic refractory wounds. However, not all wound repair can regain both function and appearance. Especially for some deep burns, electrical burns or thermal pressure injuries in the facet joints of functional sites such as hands and feet, irreversible dysfunction has occurred at the time of injury or during treatment, accompanied by deep tissue necrosis exposed. Thus, it is difficult to repair the wound by skin graft and to restore function even with cutaneous flap. The flap repair may also need to be reoperated for the appearance problem. In addition, there are some difficulties in the repair of some small wounds with bone necrosis exposed in non-functional sites because of the poor surrounding tissue conditions, long natural healing time and skin transplantation being inapplicable, and because the significance and cost of tissue flap transplantation for wound repair are debatable. For they cannot be repaired by simple treatment methods such as skin transplantation, and it is debatable on the value and significance of tissue flap transplantation for wound repair. In view of this, we adopted a new repair method: autologous granulation tissue and autologous thin split-thickness skin transplantation, which not only repaired the wound in a simple way but also avoided the cost of tissue flap transplantation. We have applied this method to repair more than ten cases of such wounds clinically and achieved satisfactory clinical results, The report is as follows.

## 2 Material and methods

### 2.1 Clinical information

From June 2019 to July 2022, a total of 11 patients, including 9 males and 2 females, aged 30–62 years in this group were treated. The causes of injury were: flame burn in 2 cases, boiling water scald in 1 case, electrical burn in 5 cases, and thermal crush injury in 3 cases. In 11 patients, there were 20 exposed wounds, 1*2 cm^2^-4*6 cm^2^ in size, distributed in the head, hand, wrist, calf and ankle with bone, joint and tendon necrosis.

### 2.2 Surgical methods

1) Thoroughly debride the wound. The necrotic and exposed bone tissue is removed by chisel, the completely necrotic tendon tissue is removed, and the necrotic soft tissue around the wound is completely excised until the wound surface is bleeding. We thoroughly rinse the wound and cover it with wet gauze to be transplanted.
2) Cut the granulation tissue from other parts of the patient's body until it is enough to cover the above wound. The granulation tissue with a thickness of about 0.5–0.8 mm is rinsed and soaked in physiological saline containing growth factors.
3) Cut the split skin from other parts of the body, and apply a pressure bandage to the donor area.
4) Cover the deep wound with the granulation tissue after thorough debridement, then graft the autologous split skin onto the deep wound covered with granulation tissue and other parts of the patient.
5) Pressure bandage and fixation of the surgical area.

## 3 Results

In 11 patients, 20 wounds surgically treated healed 15–25 days after the operation, and no bone tissue, joints, or tendons were exposed. In the case of exposed necrotic bone and joint, the broken end was not fixed and fused with steel pins after debridement, but only by a bandage. No case underwent secondary surgery after surgery. Some wounds were treated with bedside allograft with the consent of the patient due to a small amount of residual granulation wound healing after transplantation.

### 3.1 Typical case 1

**Fig. 1:**
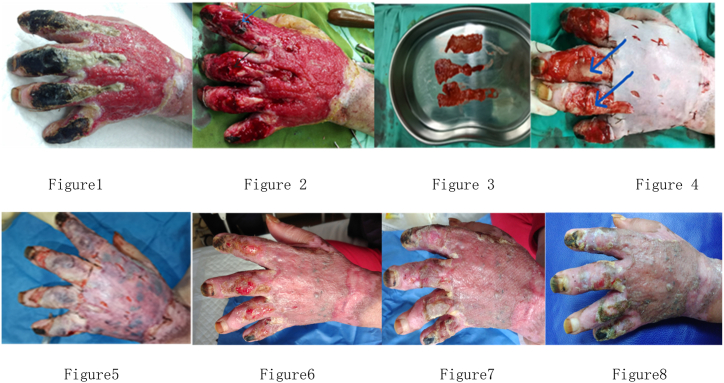
Figure 1 shows the preoperative situation. Figure 2 shows the debridement during the operation, the black arrow indicates the necrotic proximal interphalangeal joint and the cortex of the phalanx that have been bitten off, and the blue arrow indicates the necrotic proximal interphalangeal joint that has not been bitten off. Figure 3 shows the excised granulation tissue ready to cover the wound. Figure 4 shows the skin graft on the wound after debridement, and the blue arrows were the granulation tissue covering the interphalangeal joints and phalanges. Figure 5 shows 3 days after skin transplantation. Figure 6 shows 15 days after skin transplantation. Figure 7 shows 5 days after bedside allogeneic skin transplantation. Figure 8 shows 10 days after allogeneic skin transplantation.

**Fig. 2:**
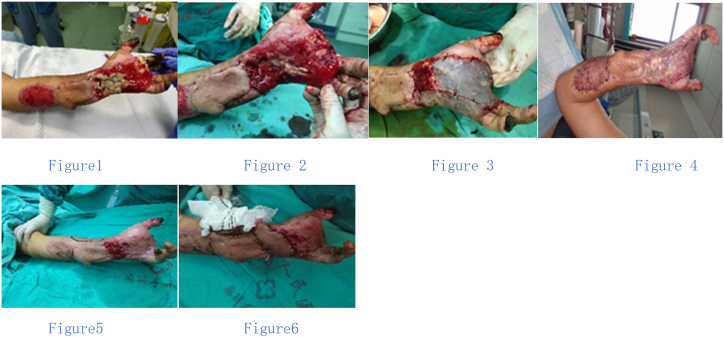
Figure 1 shows the wrist necrotic bone exposure (20 days after admission). Figure 2 shows the wrist after debridement. Figure 3 shows the granulation tissue in the donor site of the forearm flap is harvested to cover the exposed wounds (4*6 cm^2^) of the wrist bone and tendon, and some of the grafted skin covers the granulation tissue transplanted in the ulnar and distal wrist. The granulation wounds without grafted skin are covered with scalp during the operation. Figure 4 shows the survival of the skin graft in the operation area one week after the operation. Figure 5 shows the wrist debridement again for the wrist joint was unstable and the tissue at the radial artery is very weak. Figure 6 shows the flap to repair the wrist wound.

A 65-year-old male patient was scalded by boiling water on his left hand for several minutes due to an epileptic seizure. After one month of treatment in the clinic, he went to our hospital because the wound did not heal. Examination on admission: the wound on the palm was healed, the back of the hand was covered by granulation tissue, part of the extensor tendon was necrotic and exposed, the 2nd, 3rd, 4th, and 5th finger dorsal necrotic eschar was attached, necrotic tendons and bones and joints accompanied by a large amount of liquefied necrotic tissue and purulent secretions were exposed, the left hand was swollen obviously, and the interphalangeal joints were straight and dysfunctional (Fig. 1 Case 1). After admission, systemic antibiotic therapy and wound dressing treatment were performed. Surgical treatment was performed on the 5th day after admission. During the operation, the aged granulation tissue on the dorsal hand was removed, and the necrotic soft tissue and tendon tissue of the wound were excised. The dorsal phalanx was gray-yellow, and the interphalangeal joint was blue-black. The necrotic cortex of the phalanx and the necrotic interphalangeal joint were bitten off until the wound surface is bleeding (Fig. 2 Case 1). The wound surface was rinsed thoroughly and wet compressed, and the granulation tissue on the dorsal hand was re-cut with a thickness of 0.5 mm for use (Fig. 3 Case 1). The partial-thickness skin and thin split-thickness skin were excised from the outer side of the patient's upper arm, and then pressure dressings were applied at the donor site. The partial-thickness skin is transplanted to the dorsal hand, and the excised granulation tissue is first transplanted to the dorsal side of the fingers and then covered with thin split-thickness skin and fixed (Fig. 4 Case 1). Systemic antibiotic therapy was used to improve the survival rate of the flap according to the wound culture during the perioperative period. 3 days after the operation, the dressing was changed in the surgical area. Most of the skin grafts on the dorsal hand and the dorsal side of fingers survived (Fig. 5 Case 1). After the dressing change, a small amount of granulation tissue remained on the dorsal side of the fingers on the 15th day after the operation (Fig. 6 Case 1). Allograft skin grafting [1,2] was performed at the bedside with the consent of the patient (Fig. 7 Case 1). The wound healed and the patient was discharged 25 days after the operation (Fig. 8 Case 1).

### 3.2 Typical case 2

The patient was a 35-year-old male. Admission diagnosis: high voltage electric injury of the left wrist with head trauma. The patient was admitted 6 h after injury. On the same day, scalp debridement and suture, and left wrist relief incision were performed. The left wrist debridement was performed 5 days after admission. During the operation, the ulnar artery and ulnar vein were found to be embolized at the wrist, and the proximal end of the embolization was ligated. At the same time, the palmar necrotic tendon of the wrist was removed, and the wrist was covered with a local flap. After the operation, the distal end of the flap and the wrist tissue were cracked, and some wrist tissue was necrotic and exposed. Wrist necrotic tissue removal was performed 20 days after admission (Fig. 1 Case 2). Most of the necrotic tissue including part of the necrotic carpal bone was removed during the operation (Fig. 2 Case 2). The wrist wound (4*6 cm) was covered with granulation tissue cut from the forearm flap area, and autologous scalp transplantation was applied (Fig. 3 Case 2). Most of the skin grafts survived after the operation (Fig. 4 Case 2). However, the stability of the wrist joint was poor after the operation. The wrist was debrided again on the 35th day after admission (Fig. 5 Case 2). The radial artery perforator flap was used to repair the wrist wound (Fig. 6 Case 2). One week after the operation, the patient was discharged from the hospital with wound healing.

## 4 Discussion

In the early stage of skin wound repair, it is often accompanied by the formation of a new matrix, usually called granulation tissue, which usually begins to form about 4 days after the injury. The newly formed capillaries make up the new matrix, the organization of which takes on a granular appearance upon cutting and visual inspection. Macrophages, fibroblasts, and blood vessels enter the wound space as a unit, and granulation tissue is essential for wound healing, filled with new capillaries and connective tissue. New capillaries and endothelial cells contribute to the formation of new blood vessels. This process ensures the supply of nutrients to the new granulation tissue [3]. As adult skin wounds heal, a period of rapid and robust capillary growth creates a vascular bed with more capillaries than normal tissue [4]. A well-known feature of the wound-healing process is the ingrowth of new capillaries or angiogenesis. At its peak, the capillary content in healing wounds maybe three or more times that of normal tissue [5]. In this study, the wounds in the recipient area were thoroughly debridement until part of the wounds bleed, and the granulation tissue was transplanted to quickly establish blood supply, so as to achieve the purpose of repairing the wounds. In this way, the connection between the recipient area and the graft vasculature can be established naturally and quickly [6], and the purpose of rapidly restoring tissue perfusion to repair the wound can be achieved. Previous studies have shown that complete vascularization of 3-mm tissue constructs upon implantation takes approximately 1–2 weeks, during which time cells within the construct cannot (adequately) supply nutrients and experience hypoxia [7]. Using this method only requires the grafting of granulation tissue to the wound surface, allowing fusion of the recipient blood vessels with the vasculature in the implanted granulation tissue, which will shorten the time required to vascularize the implant, thereby avoiding vascular phases and reducing the duration of hypoxia [8]. The method of transplanting autologous granulation tissue to repair refractory wounds quickly and effectively is to take advantage of the vascularization phenomenon of autologous tissue in the process of wound repair (there is also a rich capillary network in the granulation tissue). Such vascularized tissue can better establish blood supply with the soft tissue section around the wound and the exposed bone tissue after thorough debridement, thereby ensuring the survival of the granulation tissue after transplantation and covering the wound quickly and effectively.

In clinical application, we found that some wounds had superficial tissue necrosis in the central area of the graft. Therefore, we believe that the source of blood supply for the transplanted living tissue is more likely to come from the healthier soft tissue sections around the wound. In addition, further research is needed on the thickness of the excision and the optimal time for transplantation during the formation of granulation tissue. But this study also has certain limitations: 1) the patient must have multiple wounds at the same time, When repairing deep and difficult-to-heal wounds, there must be applicable granulation wounds in other parts, The problem of autologous tissue vascularization for wound repair is still under study when there is no other wound granulation tissue available; 2) For exposed wounds with necrotic bone and joints, small joint wounds should be selected for repair. When applied to the repair of larger joint wounds, it may lead to poor joint stability after repair; 3) At present, this method is applied to repair wounds in small areas, while the possibility of using it to repair injuries in large areas is still being explored.

## 5 Conclusions

As a supplementary choice for tissue flap transplantation, autologous granulation tissue and autologous thin split-thickness skin transplantation were used to repair some special wounds can not only repair the wounds simply and effectively but also avoid the cost of tissue flap transplantation.

## Funding information

This is a clinical study, so there is no special funding to support it.

## Author contribution statement

Jun Liu: Conceived and designed the experiments; Performed the experiments; Analyzed and interpreted the data; Contributed reagents, materials, analysis tools or data; Wrote the paper.

## Data availability statement

No data was used for the research described in the article.

## Declaration of competing interest

The authors declare that they have no conflict of interest. All procedures performed in studies involving human participants were in accordance with the ethical standards of the institutional and national research committee and with the 1964 Helsinki declaration and its later amendments or comparable ethical standards. Informed consent was obtained from all individual participants included in the study. The study was approved by the local ethical committee.
